# Seroepidemiological assessment of the spread of SARS-CoV-2 among 25 and 28 year-old adult women in Finland between March 2020-June 2022

**DOI:** 10.1371/journal.pone.0305285

**Published:** 2024-07-11

**Authors:** Penelope Gray, Tiina Eriksson, Lovisa Skoglund, Camilla Lagheden, Ceke Hellström, Elisa Pin, Anna Suomenrinne-Nordvik, Ville N. Pimenoff, Peter Nilsson, Joakim Dillner, Matti Lehtinen

**Affiliations:** 1 Center for Cervical Cancer Elimination, Department of Clinical Science, Intervention and Technology, Karolinska Institutet, Stockholm, Sweden; 2 Tampere University Hospital, Tampere, Finland; 3 Division of Affinity Proteomics Department of Protein Science KTH Royal Institute of Technology, SciLifeLab, Stockholm, Sweden; 4 Infectious Disease Control and Vaccinations Unit, Department of Health Security, Finnish Institute for Health and Welfare, Helsinki, Finland; 5 Unit of Population Health, Faculty of Medicine, University of Oulu, Oulu, Finland; 6 Biobank Borealis of Northern Finland, University of Oulu, Oulu, Finland; University of Naples Federico II: Universita degli Studi di Napoli Federico II, ITALY

## Abstract

**Introduction:**

Serological surveys of the prevalence of SARS-CoV-2 are instrumental to understanding the course of the COVID-19 epidemic. We evaluate the seroprevalence of SARS-CoV-2 among young adult Finnish females residing in 25 communities all over Finland from 2020 until 2022.

**Methods:**

Between 1^st^ March 2020 and 30^th^ June 2022, 3589 blood samples were collected from 3583 women born in 1992–95 when aged 25 or 28 years old attending the follow-up of an ongoing population-based trial of cervical screening strategies. The crude and population standardized SARS-CoV-2 seroprevalence was measured using nucleocapsid (induced by infection) and spike wild-type (WT) protein (induced both by infection and by vaccination) antigens over time and stratified by place of residence (inside or outside the Helsinki metropolitan region).

**Results:**

During 2020 (before vaccinations), spike-WT and nucleocapsid IgG antibodies followed each other closely, at very low levels (<5%). Spike-WT seropositivity increased rapidly concomitant with mass vaccinations in 2021 and reached 96.3% in the 2^nd^ quartile of 2022. Antibodies to nucleocapsid IgG remained relatively infrequent throughput 2020–2021, increasing rapidly in the 1^st^ and 2^nd^ quartiles of 2022 (to 19.7% and 56.6% respectively). The nucleocapsid IgG seropositivity increased more profoundly in participants residing in the Helsinki metropolitan region (4.5%, 8.4% and 43.9% in 2020, 2021 and 2022 respectively) compared to those residing in communities outside the capital region (4.5%, 4.3% and 34.7%).

**Conclusions:**

Low SARS-CoV-2 infection-related seroprevalence during 2020–2021 suggest a comparatively successful infection control. Antibodies to the SARS-CoV-2 WT spike protein became extremely common among young women by the end of 2021, in line with the high uptake of SARS-CoV-2 vaccination. Finally, the rapid increase of seroprevalences to the SARS-CoV-2 nucleocapsid protein during the first and second quartile of 2022, imply a high incidence of infections with SARS-CoV-2 variants able to escape vaccine-induced protection.

## Introduction

The known spread of SARS-CoV-2 through Europe started in Spain and Italy at the beginning of 2020 [1, 2]. The first known case in Finland was diagnosed in Lapland on the 29^th^ of January 2020 in a person who had recently travelled from Wuhan [3]. Finland quickly adopted health policies aimed to contain the SARS-CoV-2 epidemic and to allow the national health services to prepare for emergency health care necessary for an influx of COVID-19 cases [4, 5]. During the first months of the epidemic Finland implemented stringent restrictions, adopting the identification/contact tracing/isolation (ITI) strategy, which was reflected in low COVID-19 case numbers [5]. During the early phase of the pandemic in Finland with stringent ITI policies in place, the effective reproduction number, *R*, of SARS-CoV-2 was estimated to be well below 1 until the end of Summer 2020 when it increased to 1.1–1.4 in the Autumn, before falling below 1 again in the end of the year [5]. In 2021, the estimated *R* in Finland was between 1 and 1.3 until mid-March, after which it remained below 1 until the beginning of Summer, when it increased to almost 1.5 [5].

Following the initial first peak of SARS-CoV-2 in Finland between March and May in 2020, the number of new confirmed COVID-19 cases remained below 100 per 100,000 inhabitants until Autumn 2020 [6–8]. Thereafter there were four distinct peaks in the incidence of SARS-CoV-2 in Finland: from September to November 2020, from February to April 2021, and subsequently those caused by the Delta and Omicron variants of SARS-CoV-2 starting in September 2021 and December 2021, respectively [5]. The greatest incidence of new registered COVID-19 occurred in Finland in January to April 2022, coinciding with the dominance of SARS-CoV-2 Omicron BA.1 and later Omicron BA.2 variants in Finnish wastewater surveillance [5, 7]. The cumulative incidence of registered COVID-19 cases in Finland was reported to be the highest among people aged 20–39 years-old between March 2020 and May 2022 [5, 7]. However, the true changes in incidence of infections over time is difficult to ascertain owing to changes in testing capacity and recommendations, legislation and not unambiguous reporting recommendations.

**Fig 1:**
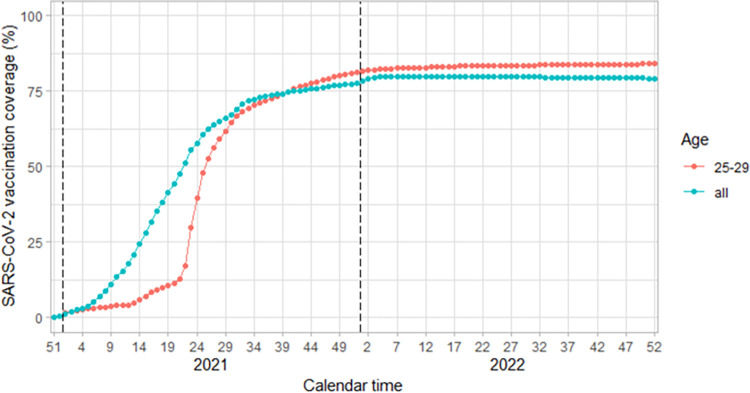
SARS-CoV-2 vaccination coverage (%) among all residents and residents aged 25 to 29 years old in Finland over time stratified by week. The vaccination coverage data is from publicly available aggregated data as last reported on the 2nd of January 2024 from the Finnish Institute of Health and Welfare [12].

Nationwide SARS-CoV-2 vaccination was commenced in Finland on the 27^th^ of December 2020, initially prioritizing healthcare workers with direct contact to COVID-19 patients, persons in long-time care facilities and healthcare workers in those facilities [9]. The vaccination campaign was then widened to subsequent COVID-19 risk strata of the population [9–11]. By the December 2021, 80% of the target population for vaccination in Finland, those over 12 years old, was reported to have received at least 1 dose of the SARS-CoV-2 vaccination, with over 80% of young adults under 30 years old having received 1 dose by the 1^st^ of June 2022 (Fig 1) [5, 12]. This was further affirmed by a study of SARS-CoV-2 seroprevalence among adults aged 18–70 years old residing Finland 2020–22 which found that over 95% of adults had detectable antibodies to the SARS-CoV-2 spike protein by 2022 [7]. In Finland, the SARS-CoV-2 vaccination program offered both the adenovirus vector-based Astra-Zeneca (Oxford) vaccine and the two mRNA vaccines from Pfizer (BNT162b2) and Moderna (mRNA-1273) with a three month gap between the administration of the first and second doses [9, 11]. A third booster dose was recommended to everyone over the age of 18 years old in Finland in December 2021 [7].

We have conducted a (repeated) cross-sectional serosurveillance study of women participating in an ongoing cervical screening study across 33 Finnish communities when they were aged 25 and 28 years old from the 1^st^ of January 2020 until the 30th of June 2022 to assess the spread of SARS-CoV-2 infections in the population over time using seroepidemiology [13].

## Materials and methods

### Study design

This study utilizes a pre-existing large prospective cohort study of Finnish females participating in a series of HPV vaccination and cervical screening trials (NCT00534638 and NCT02149030), commenced in 2007 [13, 14]. In 2007–2010, all the females who were born in 1992–1995 and who were residing in 33 specific communities in Finland were identified via the Finnish Population Registry and invited to participate in a large HPV vaccination trial [15]. The female who participated in the HPV vaccination via the HPV vaccination trial were then invited to attend a cervical cancer screening trial when they were 22, 25 and 28 years old, and comprise the cohort of Finnish females of this study [13]. From January 2019 onwards, when the participants were 25- and 28-years old they were invited by phone call to attend a study site visit, where they were also invited to donate a blood sample for an HPV serology study. From the 14^th^ of June 2020, the participants were also given the opportunity to consent to participate in a SARS-CoV-2 serosurveillance study. The women who had donated blood samples in 2020 prior to the 14^th^ of June, were contacted retrospectively and given the opportunity to consent to having their blood samples used in a SARS-CoV-2 serosurveillance study.

### Laboratory analysis

The serum samples collected from the participants were analysed for the presence and relative concentrations of IgG specific to three SARS-CoV-2 antigens using a FlexMap3D instrument (Luminex Corp) with a multiplex antigen bead array in high throughput 384-plate format at SciLifeLab in Stockholm, Sweden, as previously described [16, 17]. In short, three validated antigens were covalently coupled to colour coded magnetic beads (MagPlex, Luminex Corp); Spike wild type expressed in HEK cells, Spike S1 domain expressed in CHO cells and Nucleocapsid C terminal protein, expressed in *E*. *coli* [17]. The serum samples were diluted 1:50 and incubated with the beads. The beads were subsequently incubated with an anti-IgG secondary reagent (H10104, Invitogen) to allow the readout, which is semiquantitative continuous values. Seropositivity was defined as above the mean plus 6 (Spike) or 12 (Nucleocapsid) standard deviations of twelve standard negative control samples included in each assay. A previously reported validation study estimated the sensitivity and specificity of this assay to be 99.2% and 99.8% respectively, where seropositivity was classified as being positive for at least 2 out of 3 of the tested antigens (spike S1 domain, spike S1 domain and nucleocapsid C terminal protein) among serum samples taken at least 16 days after PCR positivity for SARS-CoV-2 [17].

### Statistical analysis

To evaluate the comparability in the characteristics of the women participating over time, the proportion of samples donated in each year was calculated stratified by community of residence, age, birth cohort and quartile. The first quartile, Q1, was defined as the 1^st^ of January until the 31^st^ of March, the second quartile, Q2, encompassed the 1^st^ of April to 30^th^ of June, the third quartile, Q3, was from 1^st^ of July to the 30^th^ of September, and the fourth quartile, Q4, was from the 1^st^ of October to 31^st^ of December, for comparability to an earlier Finnish serosurvey by Solastie et. al. [7].

The crude anti- SARS-CoV-2 nucleocapsid (N) immunoglobulin G (IgG) and anti- SARS-Cov-2 spike wild type (spike-WT) IgG seroprevalence was calculated among the participants stratified by calendar time into quartiles. Missing data was excluded from the analysis. We estimated 95% confidence intervals to the point prevalence estimates using the Agresti-Coull method [18]. To increase generalizability to the population of young adults in Finland, the SARS-CoV-2 nucleocapsid and spike-WT seroprevalence over time were calculated standardized to the average population distribution between the years 2020–2022 as reported by Statistics Finland, by weighting the community specific prevalence by the population size [19].

To take account of possible differences in the transmission dynamics in the greater capital region of Helsinki, the seroprevalence of anti-SARS-CoV-2 N and anti- spike-WT seroprevalence was calculated stratified by year and grouped quartile (quartiles 1 and 2 or quartiles 3 and 4) at the time of sample donation, and place of residence (into those residing in the greater capital region of Helsinki, and those residing outside of Helsinki). Participants residing in Helsinki, Porvoo or Hyvinkää were classified as living in the greater capital region.

The statistical analyses were conducted using R statistical software version 4.2.1 with the DescTools package version 0.99.49, lubridate package version 1.8.0, ggplot2 package version 3.3.6.

### Ethical considerations

This study was granted ethical approval from the ethical review Board of the Pirkanmaa Hospital on the 14^th^ of June 2020 (ethical approval code: R20093). Participants provided written informed or parental consent to participate in the original HPV vaccination trial, and they provided written informed consent to participate in this study of SARS-CoV-2 serology over time in Finland. Participants donating blood samples prior to the date when the ethical approval was granted, were retrospectively offered the opportunity to provide written consent to have their blood sample included in this study of SARS-CoV-2 seroprevalence.

## Results

**Fig 2:**
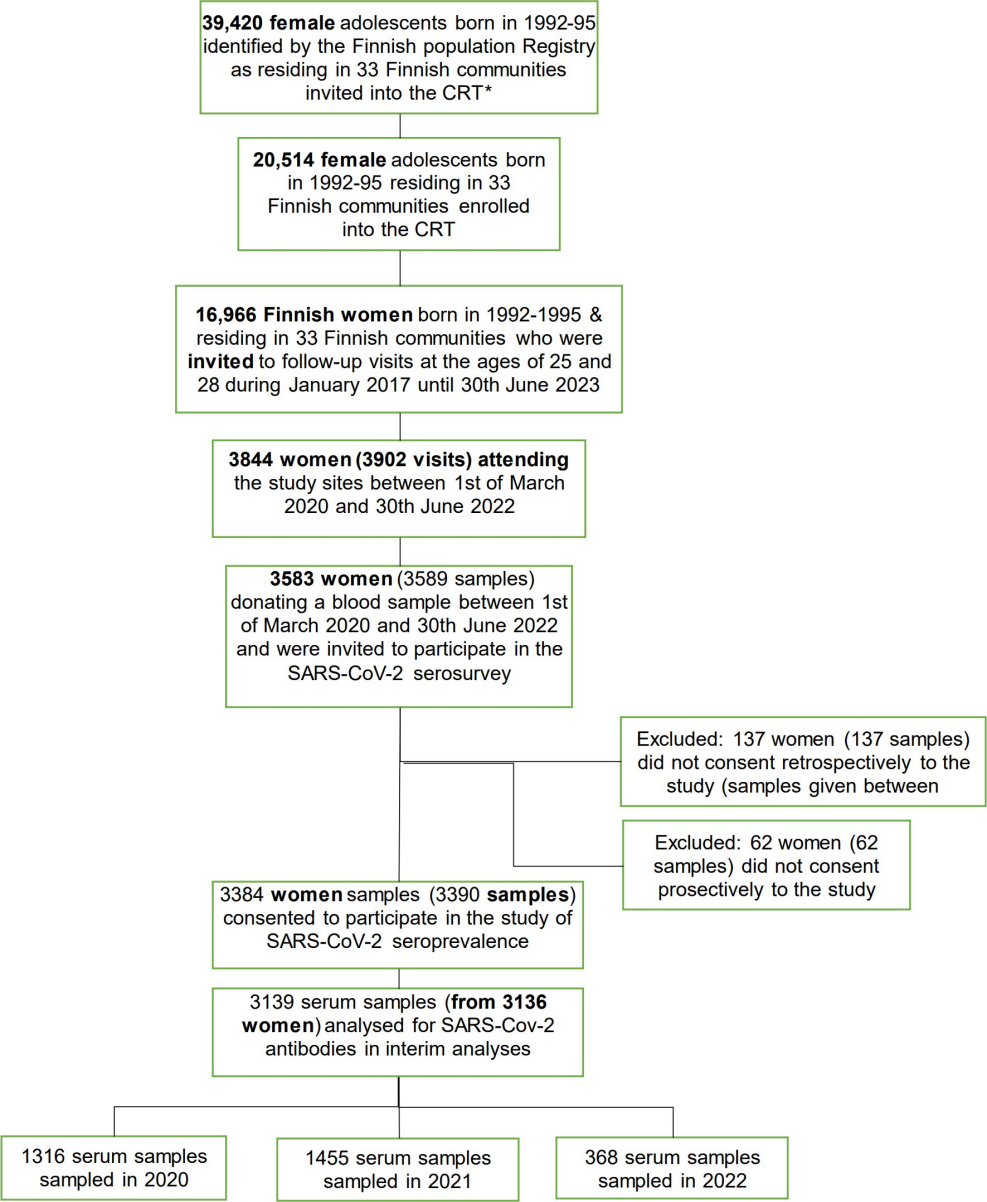
Flowchart of the study enrolment and participation. *CRT = Community Randomised HPV vaccination Trial.

Out of the 39,420 young female adolescents born in 1992–95 initially invited into the community randomized HPV vaccination trial in 2007–2010, 20,514 females (52.0%) participated. From these participants, 16,966 were invited to further follow-up at the ages of 25 and 28 years-old from 2017 until June 2024. Of these women, 6271 women participated at the ages of 25 years-old and 3771 women participated at the age of 28 years-old. Out of all the women invited to participate at the age of 25 years old 89.9%, 91.6%, 92.5% and 93.3% participated from the 1992, -93, -94 and -95 born respectively. From the women, 3844 participated between the 1^st^ of March and the 30^th^ of June 2022. Out of the 3844 participants, 3384 women (94.4%) consented to donate a blood sample. Blood samples from 3136 women were analysed for the presence of SARS-CoV-2 antibodies; 1316 samples (from 1316 women) which were donated in 2020, 1455 (from 1455 women) which were donated in 2021 and 368 (from 368 women) which were donated in 2022 (Fig 2).

Participation in the study and thereby the distribution of samples varied somewhat over time. In the first two quartiles of 2020, when participants were retrospectively invited to consent to participating in the SARS-CoV-2 serosurvey, the number of samples was lowest, 23.6% of the samples of that year. The distribution of participants from each of the 25 communities included in this study varied somewhat by year, with a smaller proportion of samples were included from the Helsinki region (the capital region) in 2022 as compared to 2020 and 2021 (Table 1). Additionally, the distribution of samples also varied slightly in comparison to the average population distribution of the communities during 2020–2022 as reported by Statistics Finland. In our study population, the proportion of participants residing in Tampere was substantially greater compared to the population average (25.2% in our study as compared to 9.1%), whilst the proportion of participants residing in the communities of Helsinki were less than the population average (18.9% in our study as compared to 24.6% respectively) (Table 1 and S1 Table).

**Table 1:** Characteristics of the study population of Finnish women aged 25 and 28 years old by year of sampling.

		Year							
		2020 (N = 1316 women)		2021 (N = 1455 women)		2022 (N = 368 women)		2020–2022 (N = 3139 women)	
Characteristic		n	%	n	%	n	%	n	%
**Quartile**	Q1	74	5.6	450	30.9	209	56.8	-	-
*** ***	Q2	237	18.0	376	25.8	159	43.2	-	-
*** ***	Q3	542	41.2	298	20.5	0	0.0	-	-
*** ***	Q4	463	35.2	331	22.7	0	0.0	-	-
**Community**	Hämeenlinna	5	0.4	9	0.6	3	0.8	17	0.5
	Helsinki	268	20.4	293	20.1	32	8.7	593	18.9
	Hyvinkää	20	1.5	23	1.6	3	0.8	46	1.5
	Joensuu	28	2.1	55	3.8	7	1.9	90	2.9
	Jyväskylä	123	9.3	101	6.9	31	8.4	255	8.1
	Kemi	40	3.0	28	1.9	3	0.8	71	2.3
	Kokkola	10	0.8	10	0.7	0	0.0	20	0.6
	Kotka	46	3.5	74	5.1	32	8.7	152	4.8
	Kouvola	16	1.2	20	1.4	8	2.2	44	1.4
	Kuopio	33	2.5	19	1.3	2	0.5	54	1.7
	Lahti	85	6.5	98	6.7	41	11.1	224	7.1
	Lappeenranta	35	2.7	31	2.1	12	3.3	78	2.5
	Mikkeli	27	2.1	19	1.3	17	4.6	63	2.0
	Oulu	39	3.0	31	2.1	7	1.9	77	2.5
	Pori	9	0.7	4	0.3	3	0.8	16	0.5
	Porvoo	24	1.8	18	1.2	6	1.6	48	1.5
	Rauma	4	0.3	3	0.2	3	0.8	10	0.3
	Rovaniemi	7	0.5	40	2.7	0	0.0	47	1.5
	Salo	11	0.8	17	1.2	5	1.4	33	1.1
	Sastamala	7	0.5	15	1.0	6	1.6	28	0.9
	Savonlinna	6	0.5	0	0.0	4	1.1	10	0.3
	Seinäjoki	32	2.4	41	2.8	7	1.9	80	2.5
	Tampere	320	24.3	348	23.9	90	24.5	758	24.1
	Turku	94	7.1	123	8.5	37	10.1	254	8.1
	Vaasa	27	2.1	35	2.4	9	2.4	71	2.3
**Age**	25-years-old	768	58.4	283	19.5	0	0.0	1051	33.5
** **	28-years-old	548	41.6	1172	80.5	368	100	2088	66.5
**Birth Cohort**	1992	547	41.6	358	24.6	6	1.6	911	29.0
** **	1993	0	0.0	814	55.9	159	43.2	973	31.0
	1994	82	6.2	9	0.6	203	55.2	294	9.4
	1995	687	52.2	274	18.8	0	0.0	961	30.6
**Missing data**									
Spike wild-type serostatus		4	0.3	0	0.0	1	0.3	5	0.2
Nucleocapsid serostatus		4	0.3	0	0.0	1	0.3	5	0.2

### Spike-WT IgG seroprevalence over time

**Fig 3:**
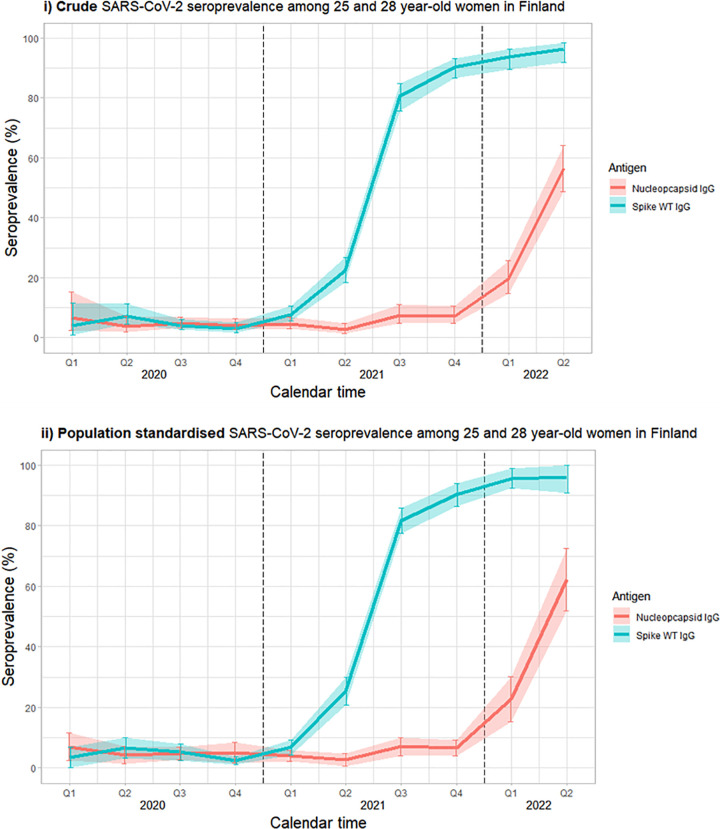
SARS-CoV-2 seroprevalence (95% confidence intervals) among Finnish women aged 25 and 28 years old from January 2020 until June 2022; i) crude SARS-CoV-2 seroprevalence and ii) population standardized SARS-CoV-2 seroprevalence according the to the community size between 2020–2022 as reported by Statistics Finland. Calendar time is divided into quartiles (Q1 = January-March, Q2 = April-June, Q3 = July-September, Q4 = October-December), vertical dashed lines indicate the start of a new calendar year.

The crude SARS-CoV-2 spike-WT type IgG seropositivity was low at 2.1% in the first quartile of 2020, increasing to 7.2 (4.5–11.3) in the second quartile of 2020 and remaining low for the two last quartiles of 2020 (Fig 3, S2 Table). The crude spike-WT IgG seropositivity started to gradually increase in the first quartile of 2021 to 7.8% (95% confidence intervals, CI 5.6–10.7%), before more rapidly increasing in the second, third and fourth quartiles of 2021, to 22.3% (95% CI 18.4–26.8%), 80.5% (95% CI 75.7–84.7%) and 90.3% (95% CI 86.6–93.1%) respectively. Finally, the crude spike-WT IgG seropositivity reached 96.2% (95% CI 91.8–98.4%) in the second quartile of 2022.

The population standardised SARS-CoV-2 spike-WT IgG seropositivity estimates over time were very similar to the crude spike-WT IgG (Fig 3, S3 Table). Although some of the estimates were slightly higher, i.e. the population standardised spike-WT IgG seropositivity was 25.3% (95% CI 20.7–30.2%) in the second quartile of 2021 as compared to 22.3% (95% CI 18.4–26.8%) in the crude estimate, the confidence intervals of the population standardised estimates overlapped the crude estimates. (Fig 3)

### N-IgG seroprevalence over time

The crude SARS-CoV-2 N-IgG seropositivity was slightly elevated in the first quartile of 2020 at 6.7% (95% CI 2.6–15.2%). It then remained relatively low for the remainder of 2020, at 3.8% (95% CI 1.9–7.2%) in Q2 of 2020, 4.8% (95% CI 3.3–7.0%) in Q3 of 2020 and 4.1% (95% CI 2.6–6.4%) in Q4 of 2020, and also in the first two quartiles of 2021, at 4.4% (95% CI 2.9–6.8%) in Q1 of 2021 and 2.7% (95% CI 1.4–4.9%) in Q2 of 2022. The N-IgG seroprevalence then started increase exponentially in the Autumn of 2021 until the end of the study period, with the seropositivity increasing from 7.4% (95% CI 4.9–11.0%) and 7.3% (95% CI 4.9–10.6%) in the Q3 and Q4 of 2022 respectively, to 19.7% (95% CI 14.9%-25.7%) in Q1 of 2022 and 56.6% (95% CI 48.8–64.1%) in Q2 of 2022 (Fig 3, S2 Table).

The population standardised SARS-CoV-2 N-IgG seropositivity was very similar to the crude seropositivity until the first 2 quartiles of 2022, when the population standardised N-IgG seropositivity was slightly higher at 22.7% (95% CI 15.4–30.1%) and 62.2% (95% CI 51.9–72.5%) during Q1 and Q2 respectively, although confidence intervals overlapped that of the crude seropositivity estimates (Fig 3, S3 Table).

### SARS-CoV-2 seroprevalence by place of residence

When stratifying the estimates of SARS-CoV-2 N-IgG seropositivity by place of residence, the N-IgG seroprevalence was similar among women residing in the Helsinki metropolitan region in comparison to those residing outside the capital region during 2020 (Table 2). However, in the third and fourth quartile of 2021 the N-IgG seropositivity was notably higher in participants residing in the metropolitan region where the seropositivity was 12.8% (95% CI 8.4–19.0%) compared to those residing outside the capital region where the seroprevalence was 5.5% (95% CI 3.7–8.0%) (Table 2). When stratifying further by quartile, the 2021 the N-IgG seropositivity was twice as high among the participants residing in the Helsinki metropolitan region during the 1^st^, 3^rd^ and 4^th^ quartiles of 2021 compared to seropositivity among those living outside the Helsinki metropolitan region (S4 Table). In the first and second quartile of 2022 the N-IgG seroprevalence increased to 43.9% (95% CI 29.9–59.0%) among the participants residing in capital region and 34.7% (95% CI 29.7–40.0%) in the participants residing outside the metropolitan region.

**Table 2:** SARS-CoV-2 seroprevalence over time stratified by whether the participant resides in the greater capital region of Helsinki* or outside the capital region. *Includes persons residing in Helsinki, Porvoo and Hyvinkää.

a)		**Nucleocapsid IgG**					
		Women residing in the greater capital region of Helsinki*			**Women residing outside the capital region**		
**Year**	**Quartile**	n	N	Seroprevalence, % (95% confidence intervals)	n	N	Seroprevalence, % (95% confidence intervals)
**2020**	**Q1-2**	1	73	1.4 (0.0–8.1)	13	237	5.5 (3.2–9.2)
	**Q3-4**	13	239	5.4 (3.1–9.2)	32	763	4.2 (3.0–5.9)
**2021**	**Q1-2**	8	178	4.5 (2.2–8.8)	22	648	3.4 (2.2–5.1)
	**Q3-4**	20	156	12.8 (8.4–19.0)	26	473	5.5 (3.7–8.0)
**2022**	**Q1-2**	18	41	43.9 (29.9–59.0)	113	326	34.7 (29.7–40.0)
b)		**Spike-WT IgG**					
		Women residing in the greater capital region of Helsinki*			**Women residing outside the capital region**		
**Year**	**Quartile**	n	N	Seroprevalence, % (95% confidence intervals)	n	N	Seroprevalence, % (95% confidence intervals)
**2020**	**Q1-2**	3	73	4.1 (0.9–11.9)	17	237	7.2 (4.5–11.3)
	**Q3-4**	11	239	4.6 (2.5–8.1)	25	763	3.3 (2.2–4.8)
**2021**	**Q1-2**	24	178	13.5 (9.2–19.3)	95	648	14.7 (12.1–17.6)
	**Q3-4**	146	156	93.6 (88.5–96.6)	393	473	83.1 (79.4–86.2)
**2022**	**Q1-2**	39	41	95.1 (83.0–99.5)	309	326	94.8 (91.8–96.8)

The estimates of SARS-CoV-2 spike-WT IgG seropositivity were similarly low during 2021 among participants residing in the greater capital region of Helsinki (spike-WT seropositivity = 4.5% [95% CI 2.6–7.5]) and those residing outside Helsinki (spike-WT seropositivity = 4.2% [95% CI 3.1–5.6]) (Table 2). The spike-WT IgG seropositivity estimates during the first and second quartiles of 2021 were similar among women residing in the greater Helsinki region as compared to women residing outside Helsinki. However, in the third and fourth quartiles of 2021 the spike-WT IgG seropositivity estimates began to diverge among the women residing in the greater Helsinki region (spike-WT seropositivity = 93.6% [95% CI 88.5–96.6%]) as compared to the women residing outside of Helsinki (spike-WT seropositivity = 83.1% [95% CI 79.4–86.2%]) (Table 2). The spike-WT seropositivity then similarly increased to 95.1% (95% CI 83.0–99.5%) and 94.8% (95% CI 91.8–96.8%) among the participants residing in the Helsinki metropolitan region and those residing outside it respectively in the first and second quartiles of 2022 (Table 2).

## Discussion

In this study we report the SARS-CoV-2 seroprevalence over time among young adults, the group among which the SARS-CoV-2 incidence was highest, who were residing in 25 communities in Finland [5]. The seroprevalence remained extremely low, below 5% of the study population until the third quartile of 2021 when a minor increase was seen, coinciding with a surge of the Delta variant in Finland [5]. Overall, the N-IgG seropositivity remained relatively low until the first quartile of 2022 when it surged to 20% of the study participants, coinciding with the replacement of the Delta variant with the more transmissible Omicron BA.1 variant in Finnish wastewater surveillance [5]. During the first half of 2022 the N-IgG seropositivity increased to 57% of the study population despite high (>80%) vaccination coverage (as also indicated by high spike protein seropositivity), as the Omicron BA.2 variant replaced the Omicron BA.1 variant as the predominant SARS-CoV-2 variant circulating in Finland [5].

As a marker of successful SARS-CoV-2 vaccination in Finland, the S-IgG seropositivity increased dramatically in 2021 from 8% in the first quartile up to 90% in 2022 and ultimately peaked at 96% among the young adult females. Eighty percent of the Finnish population aged 18–29 were reported to have received at least one dose of SARS-CoV-2 vaccination by June 2022, but the coverage may have been even higher in our consenting study participants [5]. In Finland, contrary to it’s neighbour Sweden, strong social distancing, mask-wearing policies and restrictions on international and domestic travel were adopted early in 2020 to counteract a surge in the SARS-CoV-2 incidence [5, 20, 21]. These policies and recommendations appear to have mitigated a surge in cases in Finland, during 2020. A successful risk stratified roll-out of SARS-CoV-2 vaccination (reflected in the increase in S-IgG seroprevalence during 2021) may have further supressed the R of the circulating SARS-CoV-2 variants until the more transmissible Delta and thereafter the Omicron variants replaced the wild type and alpha variants [5, 22]. Studies have also demonstrated that the BNT162b2 and mRNA-1273 vaccines which were in widespread use in Finland had lower vaccine effectiveness against the symptomatic disease caused by the SARS-CoV-2 Omicron variant than against the Delta variant, which may further have contributed to the high incidence of SARS-CoV-2 Omicron variant in early 2022 despite high vaccination coverage [23, 24].

In our study the N-IgG seropositivity among the study population increased earlier over the study period among participants residing in the Helsinki metropolitan region than among participants residing outside, similar to the reported weekly incidence of new infections and to findings from another serosurveillance study [7]. The infection induced N-IgG seropositivity in 2021 was twice as high among the young adult females residing in the metropolitan area compared to those residing elsewhere.

In the earlier SARS-CoV-2 serosurveillance study conducted among 18–85 year-old residents in the 5 major hospital districts in Finland, the N-IgG seropositivity was found to be highest among those aged 18–29, however, there was limited sample size to accurately estimate the seroprevalence stratified by year and quartile among this age strata [7]. Regardless of differences in the study population, our N-IgG and S-IgG seropositivity estimates over time follow closely the reported estimates among their population of 18–85 years old [7]. Similarly, a study conducted in Helsinki among persons undergoing routine HIV screening, reported a large increase in SARS-CoV-2 N-IgG seropositivity in the beginning of 2022, with the highest incidence being among those under 39 years old and peaking at just under 40% seropositive in February 2022 [25]. However, the ability to make comparisons between SARS-CoV-2 serological surveys are restricted owing the use of different immunoassays and varying estimates sensitivity and specificity of the said assays. In contrast, in neighbouring Sweden, where the contact network and the policies aimed to control the pandemic differed, the seroprevalence in Stockholm during the 3^rd^ quartile of 2020 was estimated to be 9.7%, substantially higher than the N-IgG or S-IgG seropositivity found in our study during the same time period [26]. Another Swedish study of SARS-CoV-2 seroprevalence among blood donors and pregnant women in the Stockholm similarly found that the SARS-CoV-2 seropositivity was substantially higher by the third and fourth quartiles of 2020 (consistently >10%), with 19% seropositive by the end of February, much higher than our estimates from Finland [27]. The severity of the SARS-CoV-2 outbreak in Sweden was also reflected in extremely high total excess mortality estimates in May 2020 [28]. In neighbouring Russia in the city of St Peterburg (less than 200km from the Finnish border), the SARS-CoV-2 S-IgG seroprevalence among residents aged 18–34 years old during the Spring of 2020 was slightly higher (11%) compared to our estimates from April to June 2020 [29]. However, our findings of increasing N-IgG seroprevalence during 2022 mirror that found in Denmark among blood donors between January and April 2022 [30].

Monitoring the true incidence of SARS-CoV-2 in a population is prone to bias. Early in the beginning of 2020 there was no organised SARS-CoV-2 testing, thereby leading to likely underreporting of the true incidence when the surveillance relies on newly diagnosed SARS-CoV-2 infections [31]. Once organised testing was up and running, a proportion of the true SARS-CoV-2 infected will still have been missing among those who were asymptomatic and did not seek testing [31]. Overtime, the proportion of asymptomatic SARS-CoV-2 carriers in the population may have fluctuated owing to the replacement of the original circulating SARS-CoV-2 variants with newer variants [32]. Furthermore, during the Omicron epidemic in Finland at the beginning of 2022, contact tracing and testing capabilities were overwhelmed, leading to a change in testing guidelines [5]. Thereby, relying on the weekly incidence of newly reported SARS-CoV-2 infections in Finland to estimate trends in the true SARS-CoV-2 incidence is prone to bias. Acknowledging the rate of vaccination, SARS-Cov-2 serological surveys can provide a more reliable picture of the true cumulative incidence of infection over time irrespective of ongoing changes in national SARS-CoV-2 policies [33].

In earlier serological surveys, the highest incidence of SARS-CoV-2 infection was found to be among young adults in Finland [7, 25]. However, earlier studies had either limited sample size among this age group or were conducted over a short period of time [7, 25]. Our study has sufficient power to accurately estimate the true SARS-CoV-2 seroprevalence among young adult females residing in Finland from the start of the pandemic until June 2022, the age group where the transmission of SARS-CoV-2 is thought to have been the highest [5].

Furthermore, this study was strengthened by the use of an immunoassay with exemplary sensitivity and specificity estimates [16, 17]. For a study population such as Finland, where the incidence of SARS-CoV-2 was low during 2020 and most of 2021, it is crucial that the assay have high specificity, to avoid overestimating the true incidence due to the reporting of false positives.

Serosurveillance surveys may suffer from a lack of generalisability to the target population when the study population is unrepresentative of a random sample of the target population [33]. Although the proportion of study participants from each community varied over time, when calculating the population standardised seroprevalence estimates over time, they did not significantly differ from the crude seroprevalence estimates over time. However, due to the timing of enrolment into the cohort utilised in this study, during 2005–2010, women who had subsequently immigrated after the initial study enrolment were not eligible for inclusion in this study. In Finland immigrants comprised approximately 9% of the total population in 2022, a sizeable proportion of the population [19]. A previous study found that SARS-CoV-2 vaccination coverage was lower among immigrants, especially those originating from Russia, Estonia and the African continent, compared to nationwide coverage estimates, thereby it is possible that our study may have underestimated true S-IgG seroprevalence [34]. Our study may be limited due to the inclusion of only one gender, however, in a meta-analysis of previous SARS-CoV-2 serological surveys the seroprevalence did not differ by gender [35]. Furthermore, our study may be limited due to the lack of data on possible confounders such as the participants socio-economic status, profession, education and other risk-taking behaviours.

Furthermore, the ability of SARS-CoV-2 serology to accurately measure the true cumulative incidence of SARS-CoV-2 infection is imperfect due the occurrence of waning and subsequent seroreversion among the majority of previously SARS-CoV-2 infected individuals 1 year since infection [36–38].

In conclusion, the SARS-CoV-2 seroprevalence remained very low during the whole of 2020 among the young adult women. Coinciding with the successful roll-out of nationwide SARS-CoV-2 vaccination the SARS-CoV-2 S-IgG seroprevalence rapidly increased to approximately 90% of the study population by the last quartile of 2021, whilst the N-IgG seroprevalence indicative of cumulative SARS-CoV-2 infection remained low. However, during 2022 as the Omicron variant replaced the Delta variant as the predominant variant in circulation there was a large epidemic of SARS-CoV-2 occurred reflected in a rapid increase in the N-IgG seroprevalence in the first and second quartiles of 2022. Furthermore, we found evidence that the incidence of SARS-CoV-2 during 2021 was twice as high among young adults residing in the Capital region. This study provides a useful insight into the SARS-CoV-2 epidemic in Finland among the age group which were reported to have the highest incidence of infection.

## Supporting information

S1 Table Population size of the study communities. Population size of the 25 communities where the participants of the study were residing as according to Statistics Finland. The population statistics were calculated on the 31st of December of each year. *of all the 25 communities listed.
(DOCX)

S2 Table Crude SARS-CoV-2 spike-WT and nucleocapsid seropositivity. Crude seropositivity (%) of SARS-CoV-2 spike wild-type and Nucleocapsid specific antibodies among the study participants between the 1st of January 202 until the 30th of June 2022.
(DOCX)

S3 Table Crude SARS-CoV-2 seropositivity stratified by place of residence. (DOCX)

S4 Table Crude SARS-CoV-2 seropositivity stratified by place of residence. Crude SARS-CoV-2 spike wild-type or nucleocapsid seropositivity (%) among the study participants over time stratified by place of residence (into the Helsinki metropolitan area or outside the Helsinki metropolitan area).
(DOCX)
