# Supplementary material for: Seroepidemiological assessment of the spread of SARS-CoV-2 among 25 and 28 year-old adult women in Finland between March 2020-June 2022
Source: PLoS One. 2024 Jul 11;19(7):e0305285. doi: 10.1371/journal.pone.0305285 (PMC11238966; doi:10.1371/journal.pone.0305285)
Supplement: S1 Table — Population size of the 25 communities where the participants of the study were residing as according to Statistics Finland. The population statistics were calculated on the 31st of December of each year. *of all the 25 communities listed. (DOCX) [file pone.0305285.s001.docx]

**Supporting Information**

**S1 File. Population size of the study communities.**

**Table S1:** Population size of the 25 communities where the participants of the study were residing as according to Statistics Finland. The population statistics were calculated on the 31^st^ of December of each year. *of all the 25 communities listed.

|  | **Population size (N) as reported on the 31^st^ of December** | | | |  |
| --- | --- | --- | --- | --- | --- |
|  | **2020** | **2021** | **2022** | **2020-2022** | Percentage of the total population of all the communities* combined (%) |
| Hämeenlinna | 67,848 | 67,971 | 68,043 | 67,954 | 2.5 |
| Helsinki | 656,920 | 658,457 | 664,028 | 659,802 | 24.6 |
| Hyvinkää | 46,576 | 46,880 | 46,797 | 46,751 | 1.7 |
| Joensuu | 76,935 | 77,261 | 77,513 | 77,236 | 2.9 |
| Jyväskylä | 143,420 | 144,473 | 145,887 | 144,593 | 5.4 |
| Kemi | 20,437 | 19,982 | 19,499 | 19,973 | 0.7 |
| Kokkola | 47,772 | 47,909 | 48,006 | 47,896 | 1.8 |
| Kotka | 51,668 | 51,241 | 50,617 | 51,175 | 1.9 |
| Kouvola | 81,187 | 80,454 | 79,429 | 80,357 | 3.0 |
| Kuopio | 120,210 | 121,543 | 122,594 | 121,449 | 4.5 |
| Lahti | 119,984 | 120,027 | 120,175 | 120,062 | 4.5 |
| Lappeenranta | 72,662 | 72,634 | 72,650 | 72,649 | 2.7 |
| Mikkeli | 52,583 | 52,122 | 51,980 | 52,228 | 2.0 |
| Oulu | 207,327 | 209,551 | 211,848 | 209,575 | 7.8 |
| Pori | 83,684 | 83,482 | 83,205 | 83,457 | 3.1 |
| Porvoo | 50,619 | 51,149 | 51,232 | 51,000 | 1.9 |
| Rauma | 39,040 | 38,959 | 38,667 | 38,889 | 1.5 |
| Rovaniemi | 63,528 | 64,180 | 64,535 | 64,081 | 2.4 |
| Salo | 51,562 | 51,400 | 50,933 | 51,298 | 1.9 |
| Sastamala | 24,052 | 23,998 | 23,734 | 23,928 | 0.9 |
| Savonlinna | 32,662 | 32,547 | 32,085 | 32,431 | 1.2 |
| Seinäjoki | 64,130 | 64,736 | 65,323 | 64,730 | 2.4 |
| Tampere | 241,009 | 244,223 | 249,009 | 244,747 | 9.1 |
| Turku | 194,391 | 195,137 | 197,900 | 195,809 | 7.3 |
| Vaasa | 67,551 | 67,615 | 67,988 | 67,718 | 2.5 |
