# Supplementary material for: Seroepidemiological assessment of the spread of SARS-CoV-2 among 25 and 28 year-old adult women in Finland between March 2020-June 2022
Source: PLoS One. 2024 Jul 11;19(7):e0305285. doi: 10.1371/journal.pone.0305285 (PMC11238966; doi:10.1371/journal.pone.0305285)
Supplement: S2 Table — Crude seropositivity (%) of SARS-CoV-2 spike wild-type and Nucleocapsid specific antibodies among the study participants between the 1st of January 202 until the 30th of June 2022. (DOCX) [file pone.0305285.s002.docx]

**Supporting Information**

**S2 File. Crude SARS-CoV-2 spike wild-type and nucleocapsid seropositivity.**

**Table S2.** Crude seropositivity (%) of SARS-CoV-2 spike wild-type and Nucleocapsid specific antibodies among the study participants between the 1^st^ of January 202 until the 30^th^ of June 2022.

|  |  | **Spike WT IgG** | | |
| --- | --- | --- | --- | --- |
| **Year** | **Quartile** | Number of samples seropositive, n | N | Seropositivity, % (95% confidence intervals) |
| **2020** | Q1 | 3 | 74 | 4.1 (0.9-11.7) |
|  | Q2 | 17 | 236 | 7.2 (4.5-11.3) |
|  | Q3 | 22 | 541 | 4.1 (2.7-6.1) |
|  | Q4 | 14 | 461 | 3.0 (1.8-5.1) |
| **2021** | Q1 | 35 | 450 | 7.8 (5.6-10.7) |
|  | Q2 | 84 | 376 | 22.3 (18.4-26.8) |
|  | Q3 | 240 | 298 | 80.5 (75.6-84.6) |
|  | Q4 | 299 | 331 | 90.3 (86.6-93.1) |
| **2022** | Q1 | 195 | 208 | 93.8 (89.5-96.4) |
|  | Q2 | 153 | 159 | 96.2 (91.8-98.4) |
|  |  | **Nucleocapsid IgG** | | |
|  |  | Number of samples seropositive, n | N | Seropositivity, % (95% confidence intervals) |
| **2020** | Q1 | 5 | 74 | 6.8 (2.6-15.2) |
|  | Q2 | 9 | 236 | 3.8 (1.9-7.2) |
|  | Q3 | 26 | 541 | 4.8 (3.3-7.0) |
|  | Q4 | 19 | 461 | 4.1 (2.6-6.4) |
| **2021** | Q1 | 20 | 450 | 4.4 (2.9-6.8) |
|  | Q2 | 10 | 376 | 2.7 (1.4-4.9) |
|  | Q3 | 22 | 298 | 7.4 (4.9-11.0) |
|  | Q4 | 24 | 331 | 7.3 (4.9-10.6) |
| **2022** | Q1 | 41 | 208 | 19.7 (14.8-25.7) |
|  | Q2 | 90 | 159 | 56.6 (48.8-64.1) |
