# Supplementary material for: Seroepidemiological assessment of the spread of SARS-CoV-2 among 25 and 28 year-old adult women in Finland between March 2020-June 2022
Source: PLoS One. 2024 Jul 11;19(7):e0305285. doi: 10.1371/journal.pone.0305285 (PMC11238966; doi:10.1371/journal.pone.0305285)
Supplement: S3 Table — (DOCX) [file pone.0305285.s003.docx]

**Supporting Information**

**S3 File: Population standardized SARS-CoV-2 spike wild-type and nucleocapsid seropositivity**

**Table S3: Population standardized SARS-CoV-2 seropositivity**

|  | | **Seropositivity, % (95 % confidence intervals)** | |
| --- | --- | --- | --- |
| **Year** | **Quartile** | **Spike WT IgG** | **Nucleocapsid IgG** |
| **2020** | **Q1** | 3.5 (0.0-7.0) | 7.0 (2.4-11.7) |
|  | **Q2** | 6.6 (3.2-10.0) | 4.4 (1.5-7.2) |
|  | **Q3** | 5.2 (2.6-7.9) | 4.8 (2.8-6.8) |
|  | **Q4** | 2.5 (1.1-3.8) | 5.1 (1.7-8.4) |
| **2021** | **Q1** | 6.8 (4.4-9.3) | 4.0 (2.1-5.8) |
|  | **Q2** | 25.3 (20.7-30.0) | 2.7 (0.6-4.9) |
|  | **Q3** | 81.6 (77.5-85.8) | 7.1 (4.2-10.1) |
|  | **Q4** | 90.2 (86.4-94.0) | 6.7 (4.0-9.3) |
| **2022** | **Q1** | 95.5 (92.2-98.9) | 22.7 (15.3-30.1) |
|  | **Q2** | 95.9 (90.8-100) | 62.2 (51.9-72.5) |
