# Supplementary material for: Seroepidemiological assessment of the spread of SARS-CoV-2 among 25 and 28 year-old adult women in Finland between March 2020-June 2022
Source: PLoS One. 2024 Jul 11;19(7):e0305285. doi: 10.1371/journal.pone.0305285 (PMC11238966; doi:10.1371/journal.pone.0305285)
Supplement: S4 Table — Crude SARS-CoV-2 spike wild-type or nucleocapsid seropositivity (%) among the study participants over time stratified by place of residence (into the Helsinki metropolitan area or outside the Helsinki metropolitan area). (DOCX) [file pone.0305285.s004.docx]

**Supporting Information**

**S4 File. Crude SARS-CoV-2 seropositivity stratified by place of residence.**

Table S4: Crude SARS-CoV-2 spike wild-type or nucleocapsid seropositivity (%) among the study participants over time stratified by place of residence (into the Helsinki metropolitan area or outside the Helsinki metropolitan area).

| a) | | **Nucleocapsid IgG** | | | | | |
| --- | --- | --- | --- | --- | --- | --- | --- |
|  |  | **Helsinki and surrounding communities (including Porvoo and Hyvinkää)** | | | **Non-Helsinki communities** | | |
| **Year** | **Quartile** | Number of samples seropositive, n | N | Seropositivity, % (95% confidence intervals) | Number of samples seropositive, n | N | Seropositivity, % (95% confidence intervals) |
| **2020** | **Q1** | 0 | 12 | 0.0 (0.0-28.2) | 5 | 62 | 8.1 (3.1-17.9) |
|  | **Q2** | 1 | 61 | 1.6 (0.0-9.6) | 8 | 175 | 4.6 (2.2-8.9) |
|  | **Q3** | 8 | 120 | 6.7 (3.2-12.8) | 18 | 421 | 4.3 (2.7-6.7) |
|  | **Q4** | 5 | 119 | 4.2 (1.6-9.7) | 14 | 342 | 4.1 (2.4-6.8) |
| **2021** | **Q1** | 7 | 104 | 6.7 (3.1-13.5) | 13 | 346 | 3.8 (2.1-6.4) |
|  | **Q2** | 1 | 74 | 1.4 (0.0-8.0) | 9 | 302 | 3.0 (1.5-5.7) |
|  | **Q3** | 9 | 81 | 11.1 (5.7-20.0) | 13 | 217 | 6.0 (3.4-10.1) |
|  | **Q4** | 11 | 75 | 14.7 (8.2-24.6) | 13 | 256 | 5.1 (2.9-8.6) |
| **2022** | **Q1** | 6 | 25 | 24.0 (11.2-43.8) | 35 | 183 | 19.1 (14.1-25.5) |
|  | **Q2** | 12 | 16 | 75.0 (50.0-90.3) | 78 | 143 | 54.5 (46.4-62.5) |
| b) | | **Spike WT IgG** | | | | | |
|  |  | **Helsinki and surrounding communities (including Porvoo and Hyvinkää)** | | | **Non-Helsinki communities** | | |
| **Year** | **Quartile** | Number of samples seropositive, n | N | Seropositivity, % (95% confidence intervals) | Number of samples seropositive, n | N | Seropositivity, % (95% confidence intervals) |
| **2020** | **Q1** | 0 | 12 | 0.0 (0.0-28.2) | 3 | 62 | 4.8 (1.1-13.8) |
|  | **Q2** | 3 | 61 | 4.9 (1.1-14.0) | 14 | 175 | 8.0 (4.7-13.1) |
|  | **Q3** | 8 | 120 | 6.7 (3.2-12.8) | 14 | 421 | 3.3 (1.9-5.6) |
|  | **Q4** | 3 | 119 | 2.5 (0.5-7.5) | 11 | 342 | 3.2 (1.7-5.7) |
| **2021** | **Q1** | 8 | 104 | 7.7 (3.7-14.7) | 27 | 346 | 7.8 (5.4-11.2) |
|  | **Q2** | 16 | 74 | 21.6 (13.7-32.4) | 68 | 302 | 22.5 (18.2-27.6) |
|  | **Q3** | 74 | 81 | 91.4 (83.0-96.0) | 166 | 217 | 76.5 (70.4-81.7) |
|  | **Q4** | 72 | 75 | 96.0 (88.4-99.1) | 227 | 256 | 88.7 (84.2-92.0) |
| **2022** | **Q1** | 24 | 25 | 96.0 (78.9-100) | 171 | 183 | 93.4 (88.8-96.3) |
|  | **Q2** | 15 | 16 | 93.8 (69.7-100) | 138 | 143 | 96.5 (91.9-98.7) |
